# 2284. SARS-CoV-2 Transmission in Utah Nursing Homes

**DOI:** 10.1093/ofid/ofad500.1906

**Published:** 2023-11-27

**Authors:** Julia Bohman, Karim Khader, Alun Thomas, Jeffrey Ferraro, Matthew H Samore

**Affiliations:** University of Utah, Salt Lake City, Utah; University of Utah, Salt Lake City, Utah; University of Utah, Salt Lake City, Utah; University of Utah, Salt Lake City, Utah; University of Utah, Salt Lake City, Utah

## Abstract

**Background:**

The goal of this study was to apply a dynamic modeling approach to assess the impact of COVID-19 control measures in nursing homes. Utah's strategy for COVID control in nursing homes included serial point prevalence surveys, triggered when a staff or resident case was detected. However, implementation of the policy varied across nursing homes.

**Methods:**

We used state-collected testing data from 70 different Utah nursing homes from March 2020 to April 2021 to estimate a facility specific transmission. Data included facility id, COVID-19 test results, number of days since the first covid test administration, and a December 2020 population census. We constructed a stochastic Reed Frost based model, using the stan library in R using the Hamiltonian Monte Carlo algorithm to estimate parameter values.

**Results:**

COVID attack rates tended to peak between 50-100 days from the onset of included data, averaging 14.2% (Figure 1). A high degree of day-to-day variability in SARS-CoV-2 test collection was observed, consistent with uneven implementation of serial point prevalence surveys. Nonetheless, we were able to achieve excellent model calibration and estimate the basic reproductive number (R0) in all included facilities. The stochastic model captured differences between facilities; the estimated facility R0 ranged from 1.4 to 2.5 (Figure 2). We did not find evidence that a delay in collection of point prevalence survey was associated with an increased R0.

**Figure:**
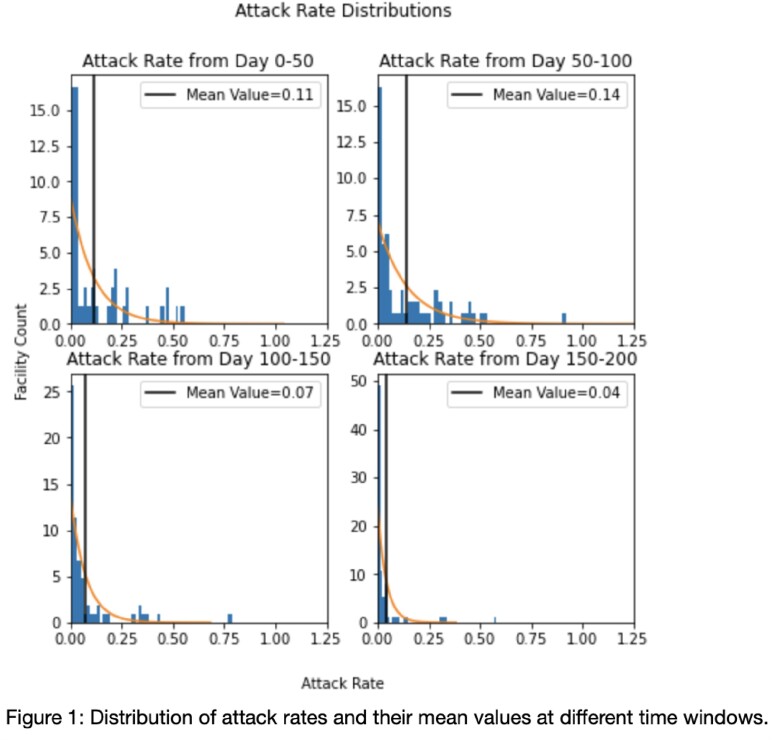

**Figure:**
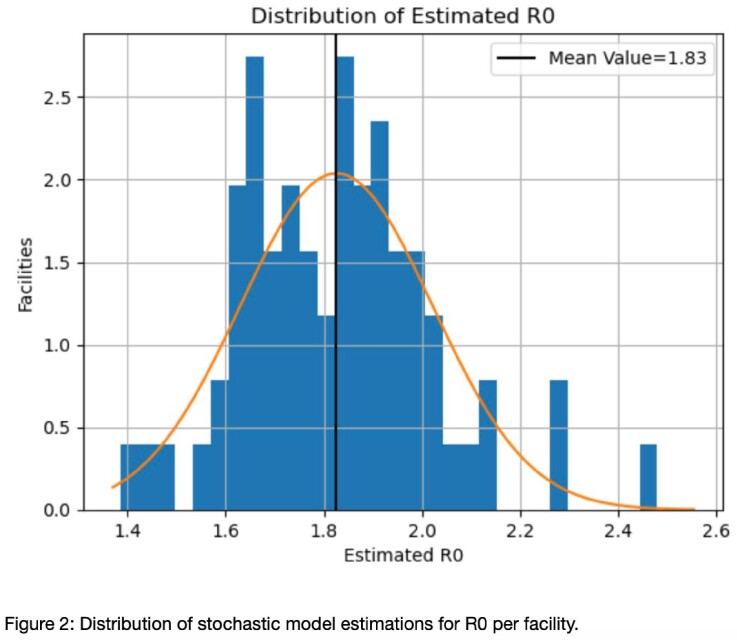

**Conclusion:**

Fitting simple dynamic models to COVID surveillance data enables a comparison of nursing homes with respect to facility R0 and an evaluation of the effect of different control policies. More complex models are needed to yield insights about the relative importance of alternative transmission pathways, such as combinations between staff and residents.

**Disclosures:**

**All Authors**: No reported disclosures

